# Psychosocial stress and stroke risk: meta-analysis of observational studies

**DOI:** 10.3389/fneur.2025.1669925

**Published:** 2025-11-06

**Authors:** Mostafa A. Khalifa, Albandari Sultan Alardhi, Ghadah Faleh S. Alharbi, Abdulmajeed Mousa M. Alzahrani, Batoul Ali Taqi, Falwah Sami Alsalman, Mohand Basher G. Albalawi, Saleh Ahmed Alzahrani, Hessah Haji Abul, Hayat Safwat Hassan, Ashwaq Dhafer N. Alqarni, Shaima ShamsEldeen KhalfAllah Ahmed, Sarah M. Hegazy, Omer Hussein Alwi Bin-Sahel, Dalia Abdalla Mohamed

**Affiliations:** 1 Faculty of Medicine, Cairo University, Cairo, Egypt; 2 Al Jahra Hospital, Al Jahra, Kuwait; 3 Department of Public Health, Al-Qassim Health Cluster, Buraydah, Saudi Arabia; 4 King Abdulaziz Medical City, Riyadh, Saudi Arabia; 5 Internal Medicine Department, Mubarak Al-kabeer Hospital, Jabriyah, Kuwait; 6 College of Medicine, Alfaisal University, Riyadh, Saudi Arabia; 7 Riyadh Second Health Cluster, Riyadh, Saudi Arabia; 8 King Salman Specialist Hospital, Hail Health Cluster, Hail, Saudi Arabia; 9 Sheikh Jaber Al-Ahmad Al-Sabah Hospital, Kuwait City, Kuwait; 10 University of Jeddah, Jeddah, Saudi Arabia; 11 Faculty of Medicine, Tabuk University, Tabuk, Saudi Arabia; 12 Faculty of Medicine, University of Khartoum, Khartoum, Sudan; 13 Faculty of Pharmacy Umm Al-Qura University, Makkah, Saudi Arabia; 14 Faculty of Medicine and Health Sciences, Seiyun University, Seiyun, Yemen; 15 Almana General Hospital, Dammam, Saudi Arabia

**Keywords:** stress, stroke, psychosocial, meta-analysis, observational studies

## Abstract

**Background:**

Psychological stress has been increasingly recognized as a potential risk factor for stroke, but the strength and consistency of this association remained uncertain. This systematic review and meta-analysis aims to determine the overall association between broad psychological stress and broad stroke risk.

**Methodology:**

Systematic searches of PubMed, Scopus, Web of Science, and OVID databases from 1990 to March of 2025. These searches utilized a strategy combining subject headings and keywords related to psychosocial risk factors and stroke. Twenty-eight studies met inclusion criteria (23 prospective cohort and 5 case-control), comprising over 950,000 participants. We excluded studies involving participants with a history of depression. Stroke was broadly defined to include ischemic, hemorrhagic, subarachnoid, TIA, and unspecified subtypes.

**Results:**

Our meta-analysis of 23 prospective cohort studies found that individuals exposed to psychological stress had a 46% higher risk of experiencing stroke (HR = 1.46; 95% CI: 1.29–1.66; *P* < 0.01). Initial analysis revealed substantial heterogeneity (*I*^2^ = 82%), which was significantly reduced to 39% (HR = 1.45; 95% CI: 1.32–1.59; *P* = 0.02) after a sensitivity analysis. Analysis of five case-control studies yielded a pooled odds ratio (OR) of 1.10 (95% CI: 1.01–1.20; *P* < 0.01), also indicating a modest but significant elevation in stroke risk; however, heterogeneity remained high (*I*^2^ = 92%). Sex-stratified analysis showed comparable increases in stroke risk for males (HR = 1.33; 95% CI: 1.19–1.49) and females (HR = 1.44; 95% CI: 1.07–1.95), with no statistically significant subgroup difference (*P* = 0.61).

**Conclusion:**

Psychological stress is significantly associated with an increased risk of fatal stroke, though publication bias and study heterogeneity highlight the need for cautious interpretation. Further research should aim to address methodological variability and selective reporting to refine our understanding of this relationship.

## Introduction

Stroke continues to be an important cause of mortality and disability in adulthood, weighing heavily on public health (1). Global estimates indicate that approximately 12 million people suffered a first-ever stroke in 2021, suggesting a rise in incidence partly attributed to the aging population and the presence of risk factors (1). Well-established risk factors for adult stroke include hypertension and smoking, diabetes, and other vascular risk factors (1). In recent years, psychosocial stress has been offered as a potential modifiable risk factor for stroke (2). Large international studies suggest that psychosocial factors might account for a large proportion of stroke risk. For instance, the INTERSTROKE case-control study attributed almost 17% of population stroke risk to psychosocial stressors (with an odds ratio of approximately 2.2 for high stress) (3). This emerging evidence outlines the impact of psychosocial environment on cardiovascular health.

Psychosocial stress arises essentially from one's interaction with social, environmental, or psychological domains. It encompasses various exposures, such as job stress, financial strain, and emotional distress due to major life events (4), Common stressors include job strain, social isolation, caregiver stress, and interpersonal conflicts (5). The stressors act on the hypothalamic-pituitary-adrenal axis to trigger cortisol, inflammatory markers, and sympathetic activity, implicating them in vascular dysfunction (6). A biologically plausible mechanism for stress contributing to stroke is its ability to raise blood pressure, enhance platelet aggregation, and accelerate atherosclerosis (7). As further mechanisms, it is also likely to increase adverse behavior, such as smoking, poor diet, and limited physical activity, thus indirectly increasing stroke risk. The recent INTERSTROKE study recognized psychosocial stress as a major risk factor for stroke, independent of confounders (3).

A meta-analysis published in 2015 synthesized results from 14 observational studies and found an overall significant positive association between psychosocial stress and risk of stroke (8). This meta-analysis found elevated odds of stroke among individuals with high stress, specifically related to general work-related stress and life event-related stress. Since 2015, many high-quality studies have emerged that were not included in earlier reviews; parameters of stress such as chronic perceived stress, occupational stress, and environmental strain were studied in diverse populations (9–13). These newer studies have addressed gaps as stroke subtypes, dose-response relationships, and stress in the absence of clinical anxiety or depression. An updated meta-analysis is therefore called for to consider this new evidence while at the same time updating the combined effect estimates and assessing heterogeneity over study designs and stress types.

It is also worth mentioning that another meta-analysis, published in 2017, found confirmation for the association across different populations and types of stress (14). However, that study had a different exclusion criteria than the one we aim to focus on. Unlike the 2017 meta-analysis, our study excludes individuals with clinical depression in a similar way to the 2015 study (8, 14). This is done in order to minimize confounding, as depression is an independent risk factor for stroke with distinct biological and behavioral mechanisms. This approach allows for a more accurate estimation of the direct association between pychosocial stress and stroke risk.

Hence, this systematic review and meta-analysis examines the expanded and most recent evidence base in order to sharpen our definition of psychosocial stress in relation to stroke risk and offer updated information to guide clinical and public health interventions. Additionally, we seek to synthesize results obtained from observational studies to aid in determining whether psychosocial stress is a significant risk factor of stroke in adults through a meta-analysis that investigates the various aspects of stress exposures (e.g., psychological, financial, work) in relation to all types of stroke, such as ischemic and hemorrhagic subtypes, as well as TIAs in the absence of depression history. Furthermore, Our study divided the participants into males and females to take into account the physiological, as well as psychological differences that determine the impact of stress on both males and females and their biological response to it (11, 15).

## Methodology

This systematic review and meta-analysis was conducted and reported in adherence to the PRISMA (Preferred Reporting Items for Systematic Reviews and Meta-Analyses) (16) and MOOSE (Meta-analyses Of Observational Studies in Epidemiology) guidelines (17).

### Study strategy

We conducted Systematic searches of published papers indexed in PubMed, Scopus, Web of Science and OVID Databases between 1990 and March 2025 using a strategy combining selected subject headings and keywords relating to psychosocial risk factors and stroke. A combination search of subject terms was applied. Subject terms included: (“psychosocial stress” OR “psychological stress” OR “mental stress” OR “work stress” OR “chronic stress” OR “job strain” OR “social stress” OR “life stress” OR “stressful life events” OR “emotional stress”) AND (“stroke” OR “cerebrovascular accident” OR “cerebral infarction” OR “ischemic stroke” OR “hemorrhagic stroke” OR “transient ischemic attack” OR “cerebral hemorrhage”) Manual searching of relevant systematic reviews and the reference lists of included studies was also conducted. Only English language studies were included.

### Study selection and eligibility criteria

Studies included in the meta-analysis met the following criteria: (1) Observational studies (prospective cohort or case–control designs). (2) Contained evidence of psychological stress exposure (e.g., self-reported psychological stress, job strain, emotional stress, and socioeconomic hardship), which were either self-reported or measured using validated tools. (3) Primary studies used adjusted models to control for confounders. Studies were excluded if: (1) they reported any history of depression (2) They were review articles, editorials, letters, or abstracts without full-text articles. Stroke was defined broadly to include ischemic, hemorrhagic, subarachnoid, TIA and unspecified subtypes. Four reviewers independently screened titles and abstracts, and bibliographic records were retrieved where available. Full-text articles of potentially relevant studies were retrieved, their eligibility was assessed by four reviewers using the predetermined inclusion criteria. Conflicts were resolved through discussion by two reviewers.

### Data extraction

Four reviewers extracted data independently using review-specific extraction tool. Data extracted included details of study design; total number of participants; inclusion and exclusion criteria; conclusion; stroke outcomes; history of co-morbidities including stroke, myocardial infarction, diabetes mellitus, hypertension, depression; type of psychosocial exposure; name of the exposure as mentioned in included studies; type of confounders in adjusted model.

### Quality appraisal and risk of bias assessment

Three reviewers independently assessed the quality of each study by using the Newcastle-Ottawa Quality Assessment Scales for Cohort Studies and Case–Control Studies. We graded the selection of participants, assessed exposure and outcome measurements, comparability and control of confounding factors. The maximum total score that could be received is 9 points.

### Data synthesis

Pooled effect estimates were calculated using random-effects meta-analysis models to account for between-study variability. For prospective cohort studies, hazard ratios (HRs) were synthesized to evaluate the association between psychological stress and stroke risk, while odds ratios (ORs) were pooled for case–control studies. Heterogeneity was assessed using the *I*^2^ statistic and Chi-square test, with values over 50% indicating substantial inconsistency. Sensitivity analysis was performed to explore the impact of individual studies on overall heterogeneity. Specifically, studies contributing to high heterogeneity were systematically excluded, and the pooled estimates were recalculated to evaluate robustness. Subgroup analyses were conducted based on sex to assess potential differences in stroke risk associated with psychological stress among males and females. Additionally, separate synthesis was performed for studies reporting on fatal stroke outcomes. Publication bias was assessed both visually using funnel plots and statistically through Egger's regression test. A significant result from this test was used to indicate the potential presence of small-study effects or selective reporting.

## Results

### Search results

**Figure 1:**
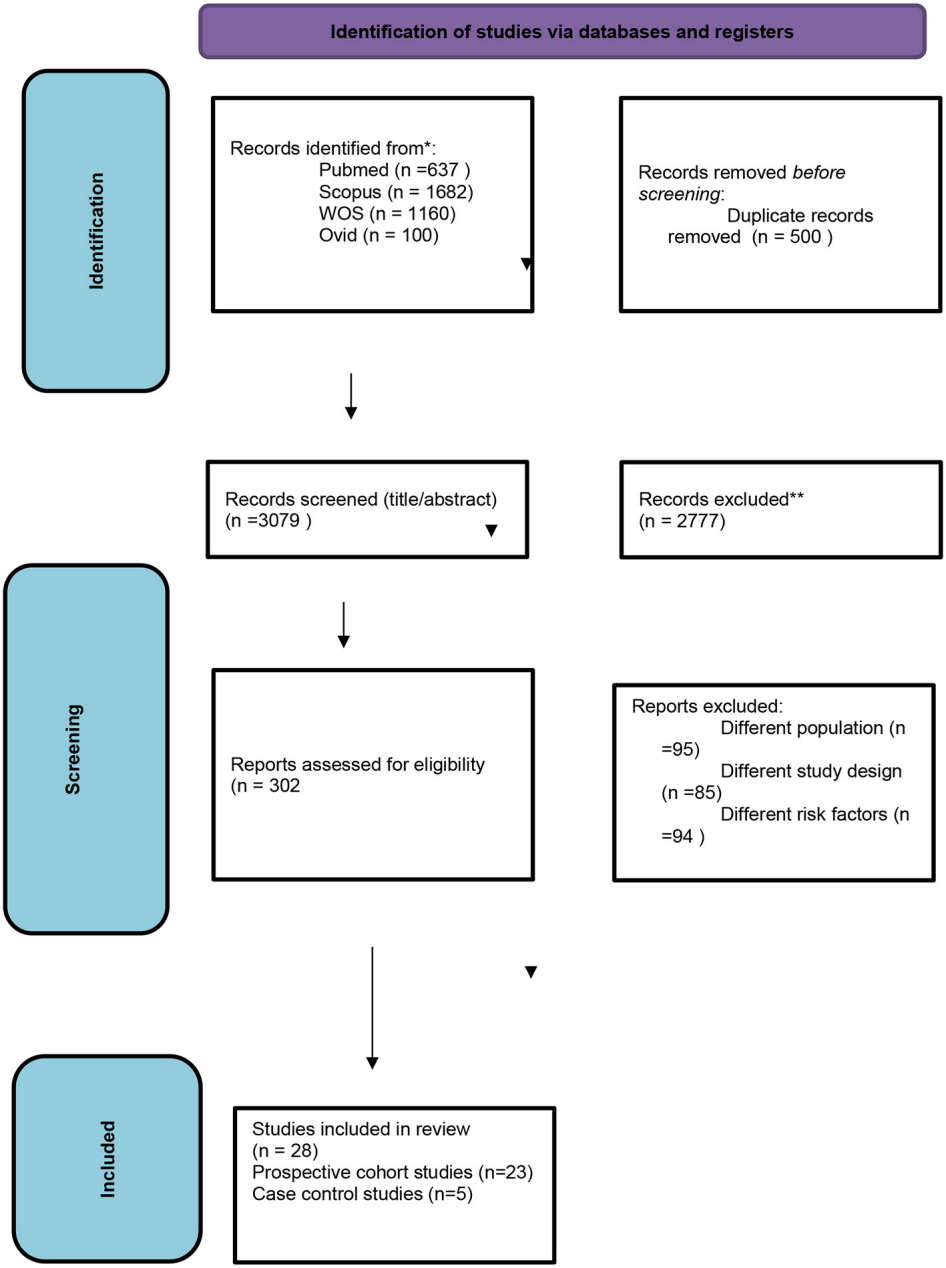
The PRISMA flow diagram.

Our primary literature search identified 3,579 records sourced as follows: 637 from PubMed, 1,160 from WOS, 1,682 from Scopus, and 100 from Ovid. Following eliminating duplicates, 3,079 articles underwent screening at the title/abstract level. After screening titles and abstracts, 302 manuscripts remained. Upon conducting the full-text screening, 274 articles were excluded, resulting in a total of 28 studies that fulfilled our inclusion criteria (Figure 1).

### Baseline characteristics

Out of the 28 studies included, 23 were prospective cohort studies and 5 were case–control studies, collectively encompassing a population of over 950,000 participants. The vast majority of these studies were conducted in Europe and Asia, with additional contributions from the United States, Russia, and Australia. Sample sizes ranged dramatically—from small, focused groups of under 400 participants [e.g., (18)] to large national cohorts exceeding 237,000 individuals [e.g., (19)].

Participants varied widely by demographic, clinical, and occupational background. Cohorts included male adolescents undergoing military conscription, older adults with diabetes, hypertensive workers, individuals with psychiatric disorders, and ethnically diverse stroke patients in case–control settings. Most studies focused on mid- to late-adulthood, though several included younger populations starting from 18 or 20 years of age.

Psychological stress exposure was defined through various lenses—occupational stress (e.g., job strain or workplace environment), emotional resilience, life satisfaction, educational or socioeconomic hardship, and psychosocial adversity. While some studies relied on single-item self-report tools, others used structured interviews or validated psychosocial scales.

Stroke outcomes included ischemic, hemorrhagic, subarachnoid hemorrhage, and total or unspecified stroke. The outcome was confirmed through medical records, imaging, national health registries, or death certificates. Ischemic stroke was the most commonly reported subtype across the studies, though many included combined or multiple stroke types (Tables 1, 2).

**Table 1:** Summary table of cohort included studies.

**Study**	**Number of participants (%male)**	**Age at baseline (y)**	**Risk Factor exposure and measure**	**Duration of follow-up (y)**	**Number of stroke events**	**Stroke outcomes**	**Risk estimates (HR; (95% CI))**	**Number of cofounders controlled for in adjusted model**
Blomstrand, 2014	1,460 women (0% male)	38, 46, 50, 54, and 60 years (five age strata)	Hypertension (≥160/≥95 mmHg or on treatment), BMI, WHR, smoking, physical inactivity, cholesterol, triglycerides, diabetes, atrial fibrillation (AF), myocardial infarction (MI), perceived mental stress, and low education	32 years	184 first-ever strokes (12.6% of cohort); 138 IS, 25 HS, 21 NS; 33 fatal	Ischaemic stroke (IS), Haemorrhagic stroke (HS), Non-specified stroke (NS), Fatal stroke (FS) defined as death within 1 month	Ischaemic stroke (multivariate): Smoking HR 1.78 (1.23–2.57), BMI HR 1.07 (1.02–1.12), Low education HR 1.17 (1.01–1.35); Haemorrhagic stroke: Physical inactivity HR 2.18 (1.04–4.58); Total stroke: Hypertension HR 1.45 (1.02–2.08); BP Grade 3 (≥180 mmHg): HR 2.73 (1.62–4.60)	9 (age, hypertension, BMI, smoking, physical inactivity, cholesterol, triglycerides, mental stress, education)
Honjo et al., 2008	20,543 women (0% male)	40–59 years; mean age: 49	Educational level (junior high, high school, college+); roles at work and home; covariates included stress, smoking, alcohol, physical activity, BMI, hypertension, diabetes, menopause	12 years	Total stroke: 451 cases (315 in JHS, 99 in HS, 37 in college); Subarachnoid hemorrhage: 81 in JHS, 21 in HS, 12 in college; Ischemic stroke: 145 in JHS, 36 in HS, 15 in college	Total stroke, subarachnoid hemorrhage, intraparenchymal hemorrhage, ischemic stroke	Total stroke (fully adjusted): JHS HR 1.49 (1.18–1.89), College HR 1.35 (0.92–1.98); Subarachnoid hemorrhage: JHS HR 2.20 (1.34–3.62), College HR 2.21 (1.08–4.51); Ischemic stroke: JHS HR 1.66 (1.14–2.74), College HR 1.49 (0.81–2.74)	10 (age, area, perceived stress, marital status, smoking, alcohol, physical activity, BMI, hypertension, diabetes, menopause)
Bergh et al., 2014	237,879 male adolescents (100% male)	Mean age: 18.4 years (range: 15–20)	Stress resilience measured during military conscription using standardized psychological interview (score 1–9)	Up to 42 years (mean 25.7 years)	3,220 first strokes (2,462 ischemic, 759 haemorrhagic)	Ischemic stroke, haemorrhagic stroke, all-stroke	All-stroke: Lowest vs. highest stress resilience HR 1.54 (95% CI: 1.42–1.67); Ischemic stroke: HR 1.51 (1.37–1.67); Haemorrhagic stroke: HR 1.54 (1.29–1.83)	7 (BMI, blood pressure, cognitive function, physical fitness, ESR, parental socioeconomic index, childhood household crowding)
Araki et al., 2004	376 elderly diabetic outpatients (33% male: 124 men, 252 women)	Mean: 75.2 years	Low wellbeing measured by the Philadelphia Geriatric Center Morale Scale (score ≤ 7); Diabetes-specific burden measured using the Elderly Diabetes Burden Scale (EDBS)	3 years	25 symptomatic strokes (24 ischemic, 1 cerebral hemorrhage)	Symptomatic stroke (primarily ischemic), neurologist-confirmed using CT or MRI	Low morale HR 3.0 (1.2–7.3); Symptom burden HR 2.6 (1.1–6.5); Social burden HR 2.9 (1.1–7.2) — all fully adjusted	Up to 13: age, sex, BMI, HbA1c, systolic BP, total cholesterol, triglycerides, HDL-C, smoking, previous IHD, duration of DM, microalbuminuria, socioeconomic variables (education, income, satisfaction with economic status, living situation)
Nielsen et al., 2005	12,111 participants (47% male)	Mean: 56 years (range: 20–93)	Alcohol intake (self-reported drinks/week) and self-reported stress (high vs low), assessed by a validated single-item stress question	Median: 15 years	608 strokes (73% ischemic, 15% hemorrhagic, 12% unspecified)	Ischemic stroke, hemorrhagic stroke, total stroke (first-ever)	Among highly stressed individuals, moderate alcohol intake (1–14 drinks/week) vs. none: RR 0.57 (95% CI: 0.31–1.07). No protective effect in low-stress individuals (RR: 1.01; 95% CI: 0.75–1.36)	9 (age, sex, education, physical activity, smoking, BMI, systolic BP, cholesterol, diabetes)
Kivimäki, 2008	3,160 male employees (100% male)	19–65 years; subgroup analysis for 19–55 years	Job strain defined by high demands and low control using the Swedish Demand–Control Questionnaire	Mean: 9.7 years	20 ischemic strokes (part of 93 total ischaemic events: MI, angina, cardiac death, stroke)	Ischaemic stroke (included in composite endpoint of ischaemic disease)	Men 19–55 years: HR 1.76 (95% CI: 1.05–2.95) for ischaemic disease; HR 1.82 (0.94–3.51) for MI/cardiac death; effect not significant in full 19–65 cohort	6–8 depending on model: age, education, salary, occupational group, smoking, physical inactivity, and biological risk factors (e.g., BP, BMI, cholesterol)
Lahti et al., 2012	12,989 (6,775 male, 6,164 women)	Participants born 1934–1944, FU from age 24–35 up to 59–70 years	Schizophrenia diagnosis (hospital records), medication use (lipid-lowering and anti-hypertensive) from national registries	35 years (1969–2004)	483 hospitalizations; 136 deaths (in total cohort)	No significant increase overall; women with schizophrenia had marginally increased stroke mortality (HR 3.84, *p* = 0.06)	Stroke mortality: HR 1.65 (95% CI: 0.52–5.20); not significant. Among women: HR 3.84 (95% CI: 0.92–15.96), *p* = 0.06	3 in base model (sex, year of birth, childhood SES); extended model added medication use
Harmsen et al., 2006	7,457 participants all male	47–55 years	Psychological stress (self-rated 5–6 on a 1–6 scale indicating persistent stress over 1–5 years), along with other clinical factors (e.g., SBP, AF, TIA, DM, etc.)	28 years (1970–1998)	1,019 first-ever stroke events	Fatal and nonfatal strokes	For psychological stress (full 28 years): HR = 1.25 (95% CI: 1.03–1.51) after multivariable adjustment	Age, SBP, antihypertensive meds, TIA, AF, diabetes, parental history of stroke/CHD, smoking, chest pain, BMI, physical activity, cholesterol, and social class
Li et al., 2008	69,625 (34,920 men) (34,705 women)	40–65 years	Socioeconomic position by: Annual income, Occupational class	10 years (1990–2000)	1,648 first-ever stroke events and 275 recurrent strokes during follow up	First ever stroke, recurrent stroke, case-fatality (28-day and 1 year mortality after first stroke reported for men only)	For annual income (lowest vs highest quartile): Men: HR: 1.29 Cl: 1.06–1.58 Women HR: 1.75 Cl 1.36-2.25	Age, marital status, country of birth, housing tenure, occupation, and education
Rosengren, 1991	6,935 (100% male)	aged 47–55	Self-perceived psychological stress, rated on a 6 point scale; scores 5-6 defined as permanent stress	11.8 years (mean)	23 stroke cases in the high-stress group; total number across entire cohort not explicitly stated	First ever stroke (fatal and non-fatal combined)	Odds Ratio (OR) = 1.8 (95% Cl: 1.1–2.8) for stroke in men with permanent stress	Age, systolic blood pressure, cholesterol, smoking, BMI, diabetes, family history of MI, occupational class, marital status, alcohol abuse (hospital record), physical activity, and stress
Kuper et al., 2007	47,942 women (0% male)	30–50 years	Years of education (proxy for SES); measured via questionnaire into 4 categories ( ≤ 9, 10–12, 13–15, ≥16 years)	11 Years	200 total strokes 121 ischemic, 47 hemorrhagic, 32 unknown	Incidence of fatal or nonfatal stroke	All strokes: HR 2.1 (1.4–2.9), *P* < 0.001 for lowest vs. highest education - Ischemic stroke: HR 2.9 (1.8–4.7), *P* < 0.001 - Hemorrhagic stroke: HR 1.4 (0.7–2.9), P = 0.35	Smoking, BMI, alcohol, diabetes, hypertension, exercise
Truelsen et al., 2003	12,574 participants 5,604 men (45%) 6,970 women (55%)	20–98 years	Self-reported stress measured by intensity (4 levels) and frequency (4 levels) via questionnaire (examples given: tension, nervousness, anxiety, sleeplessness)	13 years	929 first-ever strokes	Incidence of fatal or nonfatal stroke	Fatal stroke (high stress vs. no stress): RR 1.89 (95% CI: 1.11–3.21) Weekly stress: RR 1.49 (95% CI: 1.00–2.23) (No significant association with nonfatal stroke)	Age, sex, smoking, BMI, physical activity, systolic BP, antihypertensive treatment, alcohol intake, FEV1, history of myocardial infarction, diabetes mellitus
Ikeda et al., 2008	44,152 participants 20,985 men (47.5%) 23,167 women (52.5%)	40–69 years	Perceived social support measured by questionnaire (4 items combined into index from 0 to 5)	Average 10.7 years 1993/1994 to 2004	1,057 strokes	Incidence and mortality of stroke (327 stroke deaths recorded)	For stroke mortality (highest vs. lowest social support): - Overall: HR 1.45 (95% CI 1.00–2.10) - Men: HR 1.59 (95% CI 1.01–2.51) - Women: HR 1.25 (95% CI 0.63–2.46)	Age, smoking, alcohol, BMI, physical activity, perceived stress, occupation
Uchiyama et al., 2005	1,615 participants (56.2% male)	40–65 years	Job strain measured using simplified Karasek model:- Job demands: burden, Zcompetition, time pressure- Job control: decision-making autonomy Classified into 4 groups: low strain, passive, active, high strain	Participants were followed for an average of 5.6 years (from 1994 to 2000), with a total follow-up time of 9,087 person-years.	22 stroke events (out of 38 total CVEs)	Cerebral infarction (13 cases), Cerebral hemorrhage (6 cases), Subarachnoid hemorrhage (3 cases); most confirmed by CT scan (3 unknown)	Compared to low strain:- Active jobs: HR = 2.89 (95% Cl: 1.33–6.28) - High strain: HR = 2.45 (95% Cl: 0.87–6.93)- In women (high strain): HR = 9.05 (95% CI: 1.17–69.86)	Age, sex, SBP, BMI, total cholesterol, HDL, proteinuria, family history of stroke, LVH, ST-T changes, atrial fibrillation, smoking
Kornerup et al., 2010	9,542 participants (43% male)	Mean age was 57.9 years (56.6 in men, 59.1 in women); all participants were adults.	Major Life Events (MLE) measured via 11-item questionnaire- Events categorized into childhood and adulthood- Summed as cumulative exposure and tested for dose-response	7–10 years (from 1991–1994 baseline to 2001)	350 ischaemic strokes (after exclusions)	All strokes were validated:-233 ischaemic strokes,- 117 unspecified, Other types (e.g., hemorrhage) were excluded	Financial problems:- Childhood: HR = 1.71 (95% CI: 1.29–2.26)- Adulthood: HR = 1.60 (95% CI: 1.12-2.30) Accumulated MLEs (childhood): HR = 1.41 (95% Cl: 1.06–1.90) Accumulated MLES (adulthood): HR = 1.48 (95% CI: 1.08–2.02)	Age, sex, smoking, diabetes, physical activity, SBP, BMI, cholesterol meds, lipids, AF, cohabitation, education, income, vital exhaustion
Ohlin, 2004	13,280 (80% male)	Men: 46 years (SD 4.7); Women: 42 years (SD 8.6)	Self-reported chronic stress via questionnaire (2 items, combined stress score 0-2)	Median: 21 years	Men: 438 total strokes Fatal stroke in men: 73 Women: 41 total strokes	Fatal and non-fatal stroke Based on ICD codes	Men (fatal stroke): 2.04 (1.07–3.88) Men (any stroke): 1.25 (0.97–1.58) Women (stroke): 1.48 (0.83–2.65)	Age, family history, occupational class, marital status, smoking, alcohol, physical activity, BMI, BP, lipids
Becher et al., 2018	1,223 (44.6% male)	18–73 years	Psychosocial Safety Climate (PSC), measured by PSC-12 questionnaire	5 years (between T1 2009/2010 and T2 2014/2015)	Not reported separately for stroke	Composite outcome: Circulatory diseases (CDs), including stroke, angina, MI, and hypertension	OR = 0.98 (95% CI: 0.96–1.00) for PSC predicting CDs	Age, education, effort-reward imbalance, job strain
Harmsen et al., 1990	7,495 (100% male)	Mean 51 (range 47–55 years)	Smoking, high BP, diabetes, psychological stress, atrial fibrillation, prior TIA, intermittent claudication (measured via questionnaire, physical exam, labs)	Mean 11.8 years (1970–1983)	141 first stroke among participants	7% subarachnoid hemorrhage, 13% intracerebral hemorrhage, 42% cerebral infarction, 38% unspecified	Diastolic BP >96 mmHg: OR 2.4 (1.7–3.6); Smoking: OR 1.8 (1.2–2.6); Stress: OR 2.0 (1.3–3.2); Atrial fibrillation: OR 11.5 (4.7–28.2); Prior TIA: OR 3.5 (1.6–8.0); Intermittent claudication: OR 1.9 (1.2–3.0)	BP, smoking, diabetes, cholesterol, BMI, stress, family hx stroke, physical activity, marital status, alcohol, atrial fibrillation, prior TIA, MI hx, chest pain, dyspnea, intermittent claudication (≈12 variables)
Tsutsumi, 2009	6,553 (48.7% male)	Mean 47.5 (men), 46.8 (women)	Job strain, measured by Japanese Job Demand–Control questionnaire	Mean 11 years	147 incident strokes	Total stroke, ischemic, hemorrhagic	Men total stroke adjusted HR 2.53 (1.08–5.94)	Age, education, occupation, smoking, alcohol, physical activity, study area, plus biologic; ~7–8 confounders =
Feller et al., 2013	Women: 2,813 Men: 1,986	Unsatisfied participants mean age: 49.7 years	Life satisfaction	8 years follow up (initial data collection from 1994–1998 and follow-up for incident cases through roughly 2004–2008)	57 stroke events among participants who reported being “unsatisfied” with life at baseline (29 men + 28 women)	Low life satisfaction was linked to a higher risk of stroke in women, but not independently in men	Women HR 1.69 (LL 1.05 UL 2.73) men HR 1.40 (LL 0.89 UL 2.19)	Age, study center, smoking, alcohol intake, physical activity, education, waist-to-hip ratio, diet (fruits, vegetables, red meat, whole grain), and prevalent diseases like hypertension and diabetes
Shirai et al., 2009	3,633 men, 4,277 women	Mean age for men (50.2) mean age for women (50.1)	Perceived level of life enjoyment (self-rated on a 3-point scale: high, medium, low)	12 years (started 1990 for cohort 1 and in 1993 to 1994 for cohort 2 - ended 2005	Cases: men (153) women (99), deaths: men (61) women (30)	Men with a low level of life enjoyment had an 1.5-fold higher age-adjusted risk of stroke, The level of life enjoyment was not associated with the incidence among women.	HR for Incidence: men 1.22 (LL1.01–UL1.47) & women 1.09 (LL 0.86– UL 1.37)..... HR for Mortality men 1.75 (LL1.28–UL2.38) & women 1.06 (LL 0.69– UL 1.61)	Age, occupation, BMI, smoking, physical activity, alcohol intake, health screening, diabetes, hypertension, perceived stress, Type A personality
Schiöler et al., 2015	75,236 men	Mean age at baseline (men in their 0′s) Mean age for stroke onset 59.3	“Psychosocial work environment”	12.6 years of follow up	739 stroke event	No significant association	Not significant Highest job demand HR 1.12, LL 0.89, UL 1.40 Highest job control HR 01.09, LL 0.90, UL 1.32 Lowest social support HR 0.94, LL 0.77, UL 1.15 high strain (high demand, low control) HR 1.10, LL 0.78, UL 1.53 active (high demand, high control)] HR 1.13, LL 0.95, UL 1.34 passive (low demand, low control) HR 0.95, LL 0.73, UL 1.24 low-strain (low demand, high control): ref	Age, Age^2^ - Smoking status - Body Mass Index (BMI, BMI^2^) - Systolic blood pressure (SBP, SBP^2^)
Lin et al., 2007	Bipolar group 2,289 (female 1,214 male 1,075) appendectomy group 16,413 female 8,196 male (8,217)	Median age for the patients with bipolar disorder was 36 years, median age for those undergoing appendectomy was 34 years	Bipolar disorder, appendectomy	6 years follow up 1998–2003	Patients with bipolar disorder: 69 stroke events Patients undergoing appendectomy: 246 stroke events	Patients with bipolar disorder were twice as likely to develop stroke compared to appendectomy patients	Bipolar disorder group: Adjusted OR = 2.05 (1.73–3.54) Appendectomy group (reference group): Adjusted OR = 1.00	Demographic characteristics (age, sex, geographic region) - Medical comorbidities: hypertension, diabetes, hyperlipidemia, COPD, renal disease - Substance use: alcohol and drug dependence

**Table 2:** Summary table of case-control included studies.

**Study**	**Number of participants cases:controls (%male)**	**Age (y)**	**Cases:controls with risk factor**	**Risk factor exposure and measure**	**Stroke outcomes**	**Risk estimates (HR; (95% CI))**	**Number of cofounders controlled for in adjusted model**
Jood et al., 2009	Cases: 566 Controls: 593 % Male (both groups combined): 64%	(range 18 to 69)	Permanent self-perceived psychological stress (≥1 year): Cases: 126 (80 + 46) Controls: 46 (29 + 17)	Exposure: Self-perceived permanent psychological stress Measure: Single-item questionnaire; dichotomized to: Permanent stress (last year or last 5 years) vs. all others	Stroke type: Ischemic stroke (overall and subtypes: LVD, SVD, CE, cryptogenic) Outcome after 3 months: Death or dependency (mRS score 3–6)	Overall ischemic stroke: Adjusted OR: 3.49 (95% CI: 2.06–5.93)	Age Sex Hypertension Smoking status Diabetes Hyperlipidemia Occupational class Leisure time physical activity Waist-to-hip ratio Family history of stroke (noted separately but included in adjusted models)
Abel et al., 1999	655 cases: 1,087 controls % male cases: 44.6% % male controls: 39.9%	Between 40–90	GSRRS score (stress scale): Cases: mean 205.5 Controls: mean 206.2	Geriatric Social Readjustment Rating Scale (GSRRS) – 35-item weighted questionnaire (stressful life events in past 6 months)	First ischemic stroke	OR per 20-point increase in GSRRS: 1.01 (95% CI: 0.99–1.01)	Educational level, hypertension, cardiac disease, diabetes, socialization
Moskalenko et al., 2020	303 cases: 527 controls % male cases: 66.34% % male controls: 61.11%	Between 40–75	Presence of chronic stressors: Cases: 103 (33.99%) Controls: 187 (35.48%)	Frequent stressful situations at home/work, lack of social support, family status, socioeconomic status	Ischemic stroke on the background of hypertensive disease (HTD)	Genotype + stress: OR 1.71 (95% CI 1.00–2.92) - 5A allele rs3025058 (MMP3) → protective: OR 0.73 (95% CI 0.57–0.95)	Sex, age, BMI, BP, lipid profile, genetic polymorphisms
Egido et al., 2012	150 cases (stroke patients): 300 controls % male not explicitly mentioned in the abstract	Between 18–65 years	Exact numbers not provided; prevalence significantly higher in cases as indicated by ORs	Holmes & Rahe questionnaire (H&R) (life events) ERCTA (Type A behavior) SF12 (Quality of life) GHQ28 (General Health Questionnaire)	Occurrence of first-ever stroke	Odds Ratios (OR) since it's a case-control study H&R >150: OR 3.84 (95% CI: 1.91–7.70, *p* < 0.001) - ERCTA >24: OR 2.23 (95% CI: 1.19–4.18, *p* = 0.012)	Gender, smoking status, cardiac arrhythmia, energy drink consumption, psychological variables
Behymer et al., 2025	2,964 case/control matches, totaling 5,928 participants (41.4% female; 33.7% Black and 32.7% Hispanic)	Mean age for cases 62.1, mean age for controls 61.6	By Stress Subtypes (Median, Interquartile Range; all *p* < 0.0001) Financial stress: Cases 4 (0, 7), Controls 3 (0, 6) — OR 1.05 (1.03–1.07) Health stress: Cases 3 (0, 6), Controls 2 (0, 5) — OR 1.10 (1.08–1.12) Emotional wellbeing stress: Cases 3 (0, 6), Controls 2 (0, 5) — OR 1.13 (1.11–1.15) Family stress: Cases 3 (0, 7), Controls 2 (0, 5) — OR 1.07 (1.05–1.08) Total stress: Cases 14 (6, 23), Controls 11 (4, 18) — OR 1.04 (1.03–1.04)	Self-reported stress was rated on a scale of 0 to 10, with 0 being no stress and 10 being maximal or the highest stress level possible	ICH	Variable OR (95% CL) Financial stress 1.04 (1.01–1.06) 0.0028 Health stress 1.06 (1.03–1.08) < 0.0001 Emotional well-being stress 1.12 (1.09–1.14) < 0.0001 Family stress 1.05 (1.03–1.08) < 0.0001 Total stress 1.03 (1.02–1.04) < 0.0001	Controlled for: age, education level, medical insurance status, hypertension, hypercholesterolemia, anticoagulant use, dementia/Alzheimer's disease, alcohol use, sleep apnea risk, and body mass

### Quality assessment

In this systematic review, to ensure the accuracy of assessing the validity of the included studies, we used the Newcastle–Ottawa Scale (NOS) for the cohort and case-control studies. This scale evaluates the studies across categories: selection (up to 4 stars), comparability (up to 2 stars), and outcome (up to 3 stars). Among the 23 cohort studies, 17 were classified as low risk, 3 as moderate risk, and 3 as high risk. For the 5 case-control studies, all were low risk except one that showed moderate risk. A detailed summary of the results is provided in Tables 3, 4.

**Table 3:** Summary table of ROB assessment using the Newcastle-Ottawa Quality Assessment Scale for Cohort studies.

**Author**	**Year**	**Selection bias assessment**				**Comparability**	**Outcome**			**Assessor's overall Judgement**
		**Representativeness of the exposed cohort**	**Selection of the non-exposed cohort**	**Ascertainment of the exposure (risk factor)**	**Demonstration that outcome of interest was not present at the start of the study**	**Comparability of cohorts on the basis of the design or analysis**	**Assessment of the outcome**	**Was follow-up long enough for outcomes to occur?**	**Adequacy of follow-up of cohorts**	
Kuper et al.	2007	^*^	^*^	^*^	^*^	^**^	^*^	^*^	^*^	Low risk of bias
Truelsen et al.	2003	^*^	^*^	^*^	^*^	^**^	^*^	^*^	^*^	Low risk of bias
Ikeda et al.	2008	^*^	^*^	^*^	^*^	^**^	^*^	^*^	^*^	Low risk of bias
Lathi et al.	2008	^*^	^*^	^*^	^*^	^**^	^*^	^*^		Low risk of bias
Harmsen et al.	2006	^*^	^*^	^*^	^*^	^**^	^*^	^*^	^*^	Low risk of bias
Blomstrand et al.	2014	^*^	^*^	^*^	^*^	^**^	^*^	^*^	^*^	Low risk of bias
Rosengren et al.	1991	^*^	^*^	^*^	^*^	^**^	^*^	^*^	^*^	Low risk of bias
Li et al.	2008	^*^	^*^	^*^	^*^	^**^	^*^	^*^	^*^	Low risk of bias
Honjo et al.	2008	^*^	^*^	^*^	^*^	^**^	^*^	^*^	^*^	Low risk of bias
Bergh et al.	2014	^*^	^*^	^*^	^*^	^**^	^*^	^*^	^*^	Low risk of bias
Araki et al.	2004	^*^	^*^	^*^		^**^	^*^	^*^		Low risk of bias
Nielsen et al.	2005	^*^	^*^	^*^	^*^	^**^	^*^	^*^		Low risk of bias
Kivimäki et al.	2008	^*^	^*^	^*^	^*^	^**^	^*^	^*^	^*^	Low risk of bias
Uchiyama et al.	2005	^*^	^*^	^*^	^*^	^**^	^*^	^*^		Low risk of bias
Tsutsumi et al.	2009	^*^	^*^	^*^	^*^	^**^	^*^	^*^	^*^	Low risk of bias
Kornerup et al.	2010	^*^	^*^	^*^		^**^	^*^	^*^	^*^	Low risk of bias
Harmsen et al.	1990						^*^	^*^	^*^	High risk of bias
Becher et al.	2018	^*^			^*^	^*^				High risk of bias
Ohlin et al.	2004	^*^			^*^	^*^		^*^		Moderate risk of bias
Feller et al.	2013	^*^		^*^	^*^	^**^	^*^	^*^	^*^	Low risk of bias
Shirai et al.	2009	^*^			^*^	^**^	^*^			Moderate risk of bias
Schiöler et al.	2015				^*^		^*^	^*^		High risk of bias
Lin et al.	2007	^*^	^*^		^*^	^**^				Moderate risk of bias

**Table 4:** Summary table of ROB assessment using the Newcastle-Ottawa Quality Assessment Scale for Case-Conrol studies.

**Author**	**Year**	**Selection bias assessment**				**Comparability**	**Outcome**			**Assessor's overall Judgement**
		**Is the case definition adequate?**	**Representativeness of the cases**	**Selection of Controls**	**Definition of Controls**	**Comparability of cases and controls on the basis of the design or analysis**	**Ascertainment of exposure**	**Same method of ascertainment for cases and controls**	**Non-Response rate**	
Abel et al.	1999	^*^	^*^	^*^	^*^	^**^	^*^	^*^		Low risk of bias
Moskalenko et al.	2020	^*^			^*^	^**^	^*^	^*^		moderate risk of bias
Egido et al.	2012	^*^	^*^	^*^	^*^	^**^	^*^	^*^	^*^	Low risk of bias
Jood et al.	2009	^*^	^*^	^*^	^*^	^**^	^*^	^*^		Low risk of bias
Behymer et al.	2025	^*^	^*^	^*^	^*^	^**^		^*^		Low risk of bias

### Psychological stress

**Figure 2:**
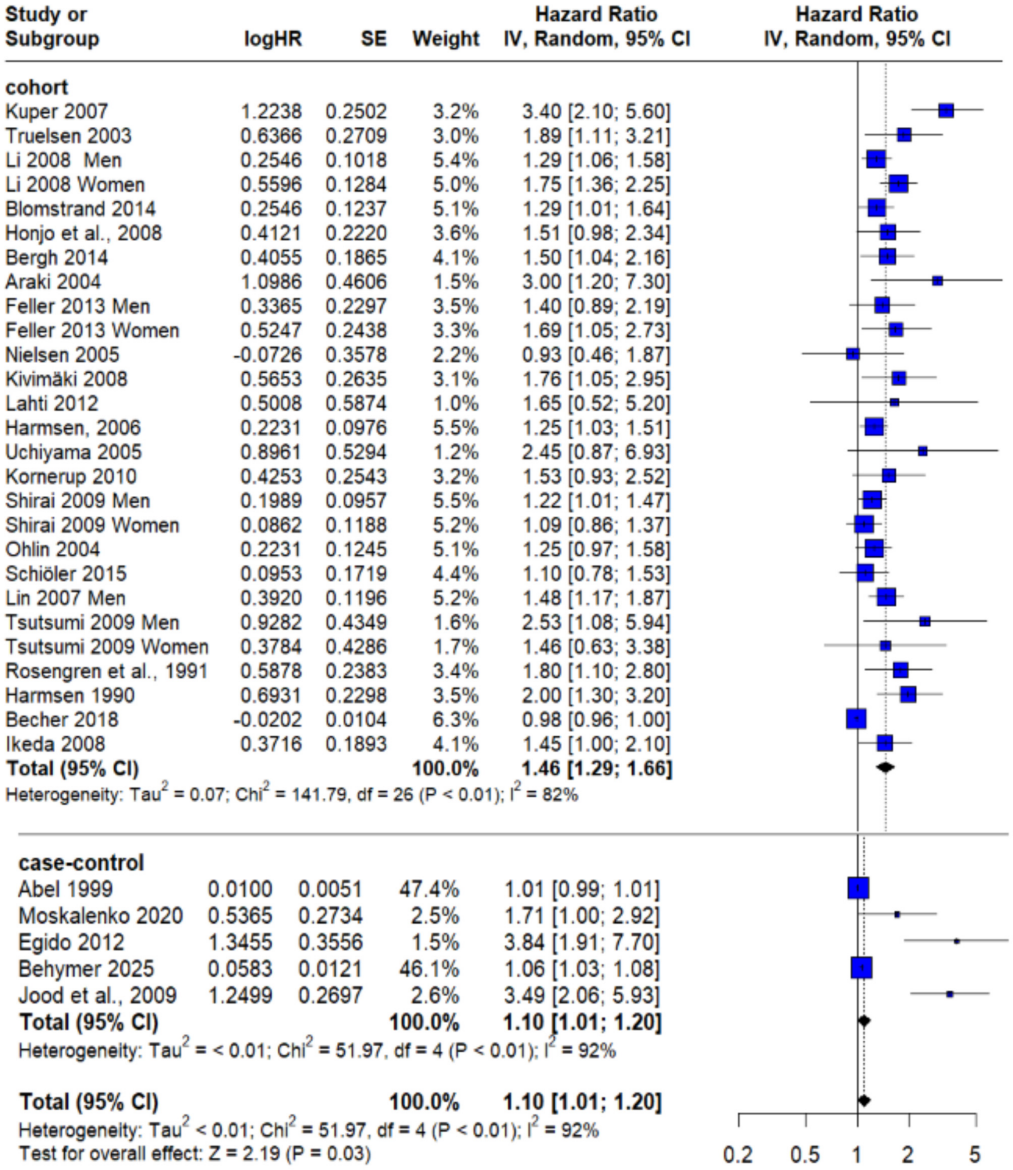
Forest plot of overall pooled effect estimate for risk of any type of stroke in subjects exposed to psychological stress.

The meta-analysis of **23 prospective cohort studies** produced a pooled hazard ratio (HR) of **1.46** (95% CI: 1.29–1.66; *P* < 0.01), indicating that individuals exposed to psychological stress had a 46% higher risk of experiencing stroke compared to those without such exposure. However, this association was accompanied by **substantial statistical heterogeneity** (*I*^2^ = 82%), as shown in Figure 2.

**Figure 3:**
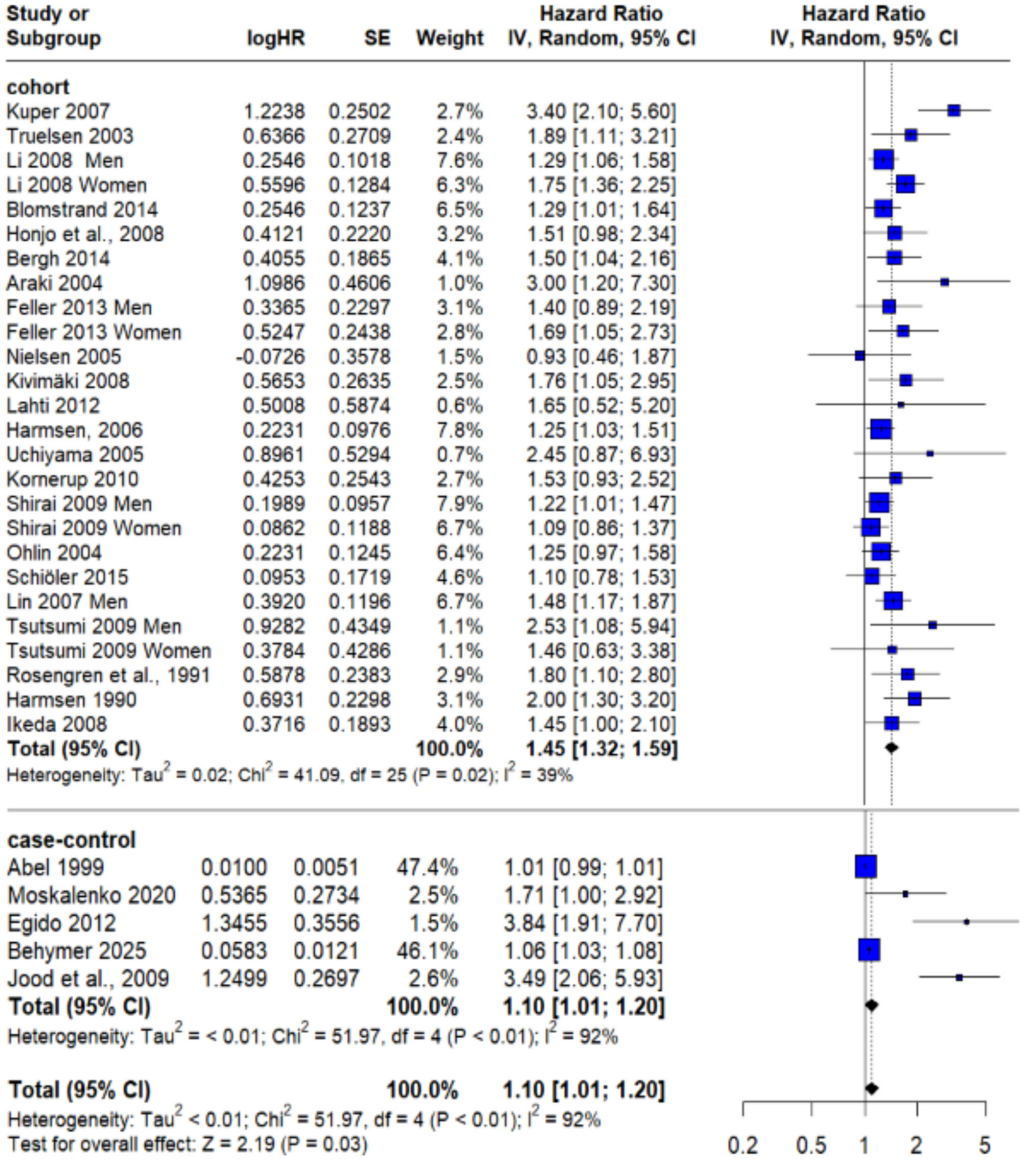
Sensitivity analysis after excluding Becher et al. (20).

To assess the robustness of this finding and identify potential sources of heterogeneity, a **sensitivity analysis** was conducted. By excluding Becher et al. (20)—which was identified as a likely contributor to variability—the recalculated pooled HR was **1.45** (95% CI: 1.32–1.59; *P* = 0.02). Importantly, this adjustment reduced heterogeneity to **39%** as demonstrated in Figure 3, thereby improving the consistency of the included studies and strengthening confidence in the observed relationship between psychological stress and stroke risk.

In parallel, analysis of **five case–control studies** yielded a pooled odds ratio (OR) of **1.10** (95% CI: 1.01–1.20; *P* < 0.01) as shown in Figure 2, also pointing to a modest but statistically significant elevation in stroke risk among individuals with psychological stress. Nonetheless, **heterogeneity remained high** in this group (*I*^2^ = 92%) and couldn't be solved.

### Stratified risk analysis by sex and stroke mortality

**Figure 4:**
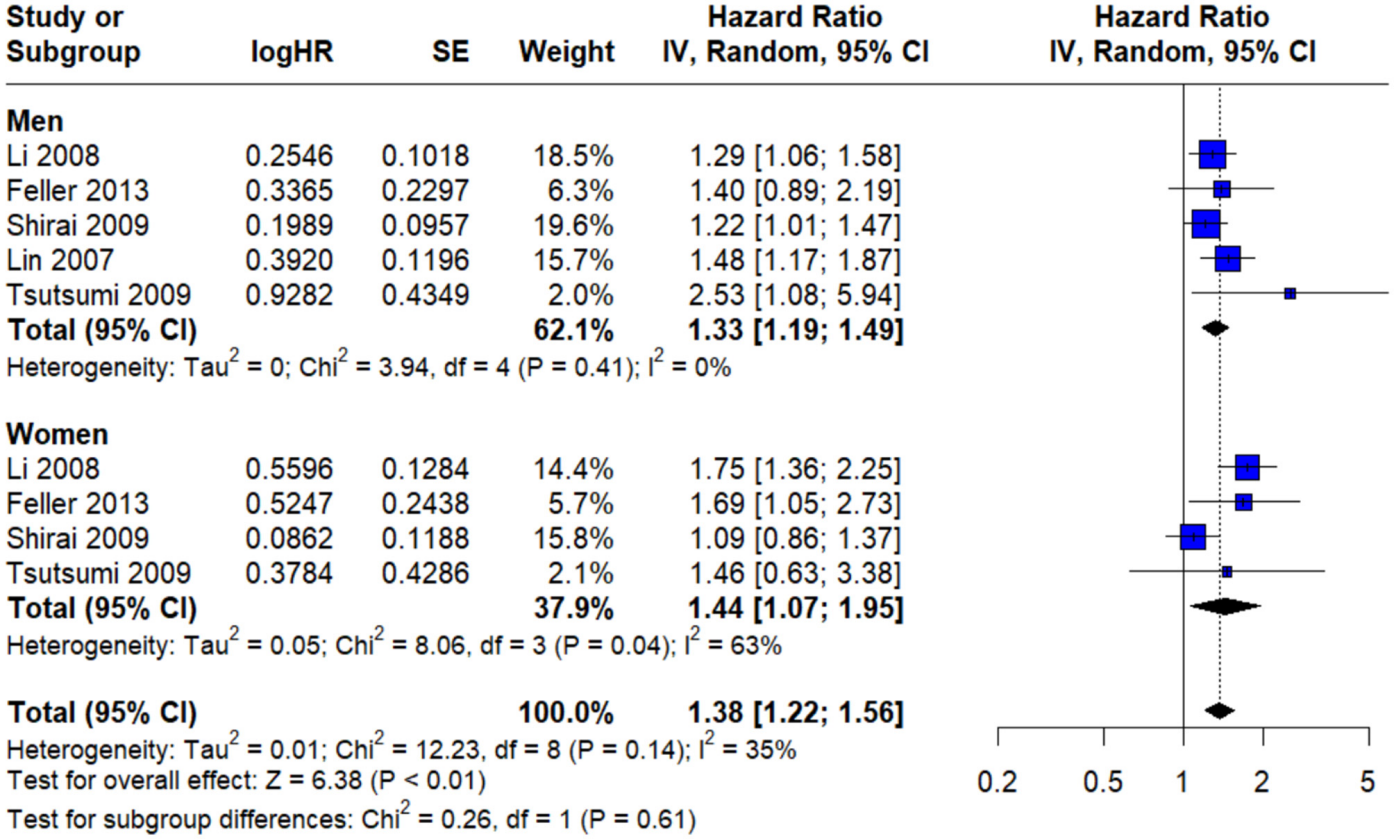
Gender subgroup allowing for a comparative analysis of the association between psychological stress and stroke risk in men and women.

Five studies provided data for **males and females separately**, allowing for a comparative analysis of the association between psychological stress and stroke risk in men and women. Among **male participants**, the pooled hazard ratio (HR) for stroke associated with psychological stress was **1.33** (95% CI: 1.19–1.49), reflecting a statistically significant increase in risk. Notably, the heterogeneity across these studies was negligible (*I*^2^ = 0%), suggesting high methodological and population consistency within this subgroup (Figure 4).

Conversely, the analysis of **female participants** yielded a pooled HR of **1.44** (95% CI: 1.07–1.95), indicating a slightly stronger association. However, this estimate was accompanied by **moderate heterogeneity** (*I*^2^ = 63%). Despite the numerical difference in risk magnitude between men and women, the **test for subgroup difference** did not reach statistical significance (*P* = 0.61), suggesting that psychological stress exerts a **comparable impact on stroke risk across males and females** (Figure 4).

**Figure 5:**
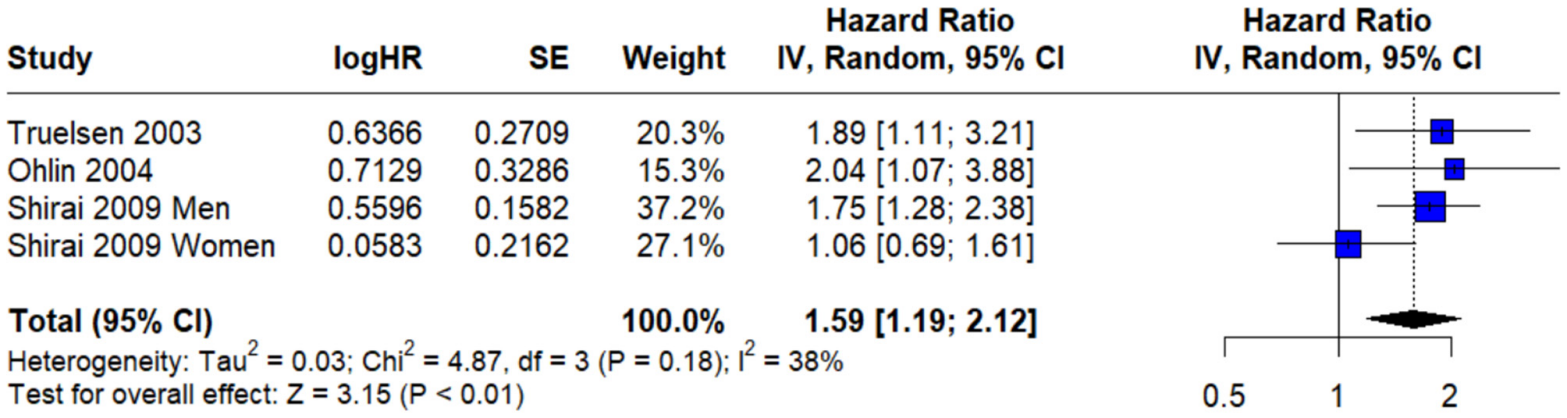
The pooled hazard ratio (HR) for fatal stroke events.

Three prospective cohort studies that specifically reported on **fatal stroke events**, the pooled hazard ratio (HR) was found to be **1.59** (95% CI: 1.19–2.12). This suggests that individuals experiencing psychological stress may have a **59% higher risk of dying from stroke**, compared to their non-stressed counterparts accompanied by **moderate heterogeneity** (*I*^2^ = 38%) (Figure 5).

### Stratified risk analysis by type of stress

**Figure 6:**
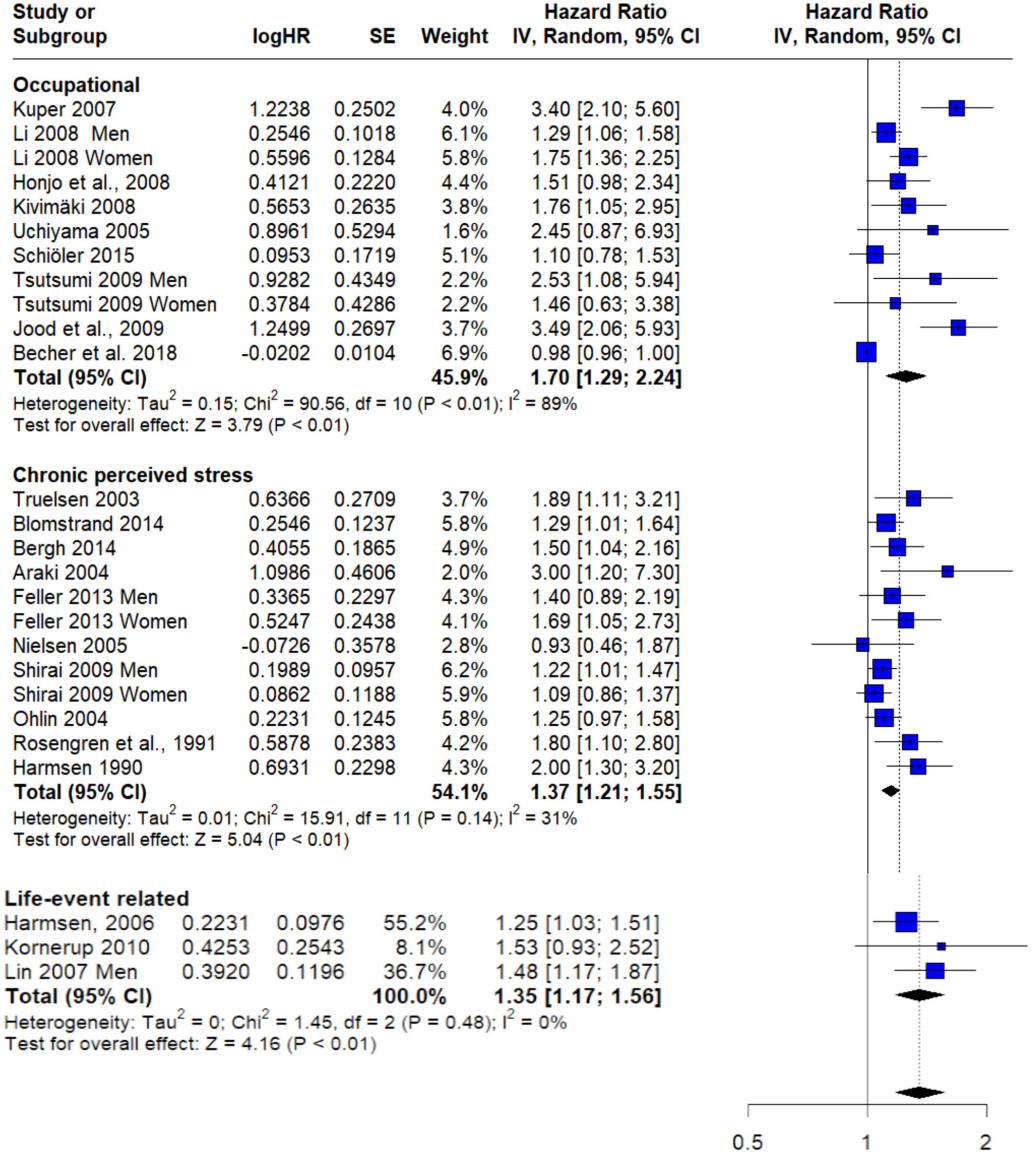
Forest plot with subgroup analysis according to stress type.

Subgroup analysis revealed a statistically significant elevated risk among individuals exposed to occupational stress, with a pooled hazard ratio (HR) of 1.70 (95% CI: 1.29–2.24; *I*^2^ = 89%). Similarly, individuals experiencing chronic perceived stress demonstrated a significantly increased risk, with a pooled HR of 1.37 (95% CI: 1.21–1.55; *I*^2^ = 31%). Life-event related stressors were also associated with a higher risk, with a pooled HR of 1.35 (95% CI: 1.17–1.56; *I*^2^ = 0%) (Figure 6).

### Co-founder adjustment

All 28 studies included in the analysis implemented multivariable models to control for potential confounding factors. Adjustment for age, smoking status, body mass index (BMI), and hypertension was reported in every study, providing a consistent baseline across the dataset.

In addition to these core variables, 25 studies adjusted for diabetes mellitus (18–40, 43), and 21 incorporated alcohol consumption as a covariate (9, 20, 22, 23, 25–27, 30–32, 34, 36, 39–41). Physical activity was included in the adjustment models of 21 studies (18, 20, 22, 23, 25–28, 30–36, 38–40), while blood lipid parameters, including serum cholesterol, were controlled for in 18 (20–23, 25–27, 30–33, 36, 38, 40, 41). These adjustments reflect a high level of methodological rigor in accounting for lifestyle and metabolic stroke risk factors.

Social determinants of health were also systematically considered. Specifically, 25 studies adjusted for socioeconomic indicators, including education, occupational status, or income level [all except (9, 40, 41)], recognizing their potential confounding influence in both psychological stress exposure and stroke risk. Additionally, 6 studies incorporated marital status or family structure as contextual variables reflecting social support (19, 20, 25, 28, 31, 35).

The consistency and breadth of confounder control across the included studies enhance the internal validity of the observed associations. While some variation in covariate selection reflects differences in study populations and data availability, the majority of studies demonstrated comprehensive methodological approaches to isolating the independent effect of psychological stress on stroke outcomes.

### Publication bias

**Figure 7:**
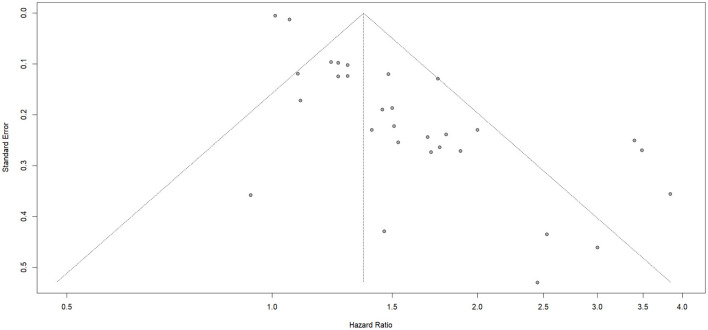
Publication bias for psychological stress.

**Figure 8:**
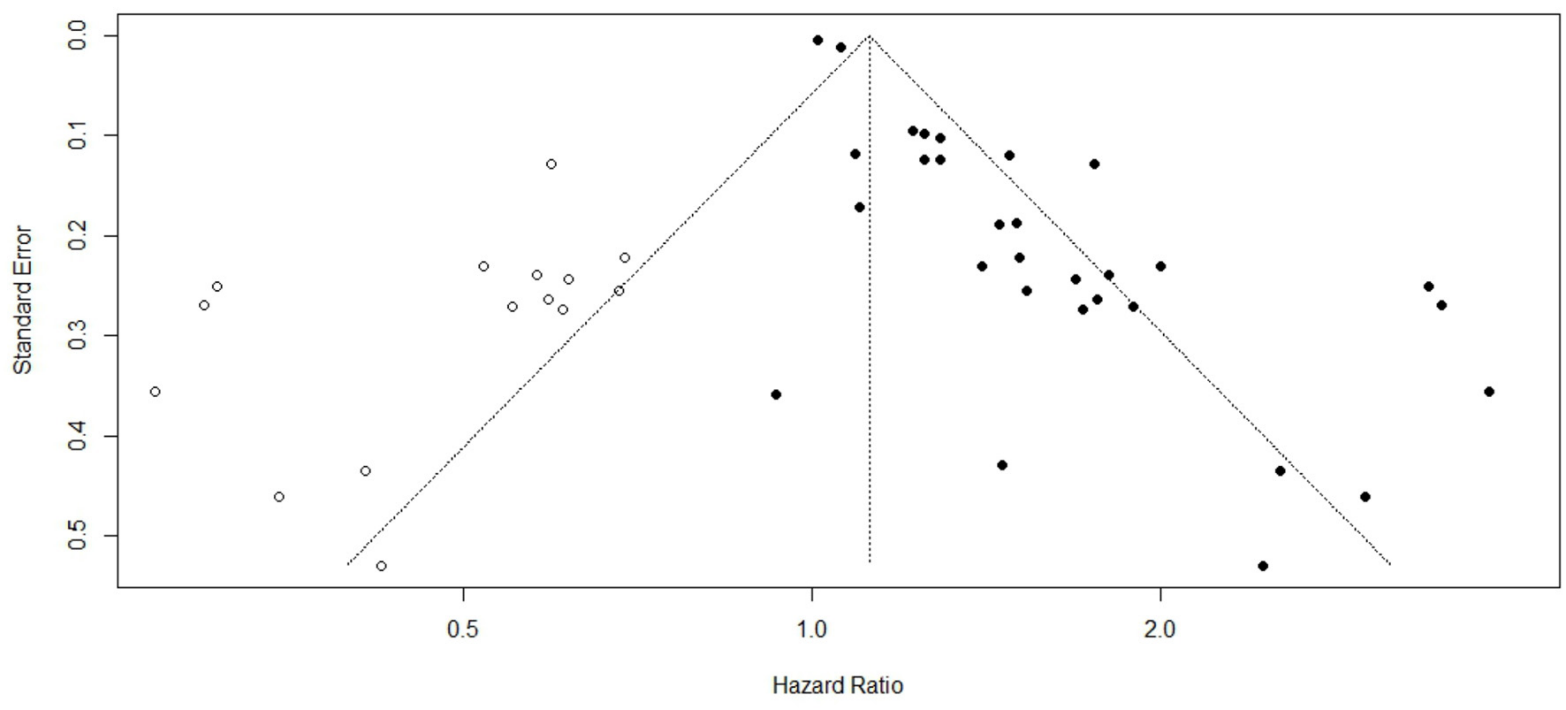
Funnel plot with trim and fill method.

The potential for publication bias was examined to evaluate the reliability and completeness of the pooled estimates. Visual inspection of the funnel plot revealed noticeable asymmetry, suggesting the possibility of small-study effects or selective publication of positive findings. In order to statistically assess this visual impression, Egger's regression test was performed. The test yielded a highly significant result (*p* < 0.0001), confirming the presence of potential publication bias across the included studies (Figure 7). This result implies that smaller or non-significant studies may be underrepresented in the meta-analysis, potentially inflating the pooled estimates. To verify the validity of the study results, correction using Duval and Tweedie's trim-and-fill method was used. The corrected pooled hazard ratio (HR) obtained using the trim-and-fill method was 1.12 (95 % CI: 1.04-1.20; *P* = 0.002), which represented a reduction from the original hazard ratio of 1.46; however, the increase in risk of stroke in individuals experiencing psychological stress still statistically significant (Figure 8).

## Discussion

Our systematic review and meta-analysis included 28 studies, 23 were prospective cohort studies and 5 were case–control studies, collectively encompassing a population of over 950,000 participants. We aim to assess the overall association between psychological stress and stroke risk across prospective cohort and case-control studies.

Our meta-analysis of prospective cohort studies found a 46% higher risk of stroke linked to psychological stress. This aligns with the findings from a recent systematic review and meta-analysis on psychosocial risk factors for stroke reported that “psychological factors” increased stroke risk by 39% (14). The consistent findings across studies strengthen the evidence that psychological stress is an independent and significant risk factor for stroke. Also, a meta-analysis using individual-level data that specifically focused on “job strain,” a widely studied measure of work-related stress, found a 24% higher risk of ischemic stroke after adjusting for age and sex (42). Given that occupational stress, including job strain, was one of the “various lenses” through which psychological stress exposure was defined in the current meta-analysis, this supports the link between work-related stressors and ischemic stroke. However, it is important to note that Becher et al. (20) was identified as a major source of heterogeneity. Therefore, a sensitivity analysis was performed after excluding this study. The observed heterogeneity can likely be explained by the relatively small sample size, the short follow-up duration, and the low incidence of circulatory diseases (i.e., stroke and myocardial infarction), which accounted for only about 8% of the total study population (20).

Conversely, the analysis of the five case-control studies included in our meta-analysis revealed a statistically significant 10% higher odds of experiencing stroke when exposed to psychological stress. However, the heterogeneity remained high in the analysis of case-control studies reflecting a weaker consistency and greater variability across studies. For example, in a case-control study (23) strong links between specific stress indicators have been found, where individuals with Holmes & Rahe values greater than 150 had 3.84 times the odds of developing an ischemic stroke and those with type A behavior had a 2.23 times the odds of having a stroke (23). Similarly, another case-control study (29), found that individuals who reported self-perceived psychological stress had 3.49 times the odds of developing an ischemic stroke (29). This observed discrepancy between the stronger associations reported in some individual case-control studies and the modest pooled effect from our meta-analysis warrants further critical examination. This could be explained by methodological variations in research design, including the vulnerability of case-control designs to recall bias, where stroke survivors may remember stressful experiences more easily than healthy controls

Given the variability in stress definitions and measurement tools across the included studies, we conducted subgroup analyses based on the type of stressor. Stress exposure was categorized into three groups: **occupational stress**, **life-event-related stress**, and **chronic perceived stress**. Our findings indicated that all stressor categories were significantly associated with stroke risk, with occupational stress showing the highest relative risk, followed by chronic perceived and life-event-related stress.

Furthermore, Our meta-analysis provided valuable sex-stratified findings, showing that psychological stress is significantly associated with increased odds of stroke in both sexes, with slight differences. Whereas for males we found a 33% higher odds of experiencing stroke when exposed to psychological stress, with no heterogeneity suggesting a consistent effect size across studies involving male participants. For females we found a 44% increased odds of stroke in the presence of psychological stress, but with moderate heterogeneity implying more variation across studies involving female participants. However, in our study the differences between the two sexes are statistically not significant, which indicate that psychological stress poses a comparable risk of stroke across sexes. Our findings are not consistent with the literature, where a meta-analysis conducted in 2015 concluded that females with perceived psychological stress have a 90% increased risk of stroke, whereas in males the risk is 24%, with females showing a greater vulnerability to stress-related stroke (8). This inconsistency between our findings and the 2015 meta-analysis could be due to differences in the effect estimates (e.g., odds ratios vs. hazard ratios), differences in the inclusion and selection criteria, or the definition and measurement of stress or the inconsistencies in the specific confounders adjusted for across studies. Furthermore, while our study suggests similar odds of stroke across sexes, we must consider the greater heterogeneity observed in female data which may have diluted the strength of the association. Also, not having a subgroup difference in our study does not exclude the possibility of sex-related association to higher vulnerability to stroke related to psychological stress.

A critical finding of our meta-analysis is the observed association between psychological stress and fatal stroke events. The pooled analysis from three prospective cohort studies indicated that individuals experiencing psychological stress had a 59% higher risk of dying from stroke (HR = 1.59; 95% CI: 1.19–2.12), with moderate heterogeneity suggesting some variability across studies. However, this result should be interpreted with caution due to the limited number of studies contributing to this analysis, which may reduce the precision and generalizability of the pooled estimate. Despite this, the direction of association aligns with previous literature. For example, a 2015 meta-analysis of 14 studies found that perceived psychological stress is associated with a 45% increased risk of fatal stroke (8) and the INTERSTROKE study reports similar findings of higher risk of all types of stroke, including fatal strokes when reporting high levels of self perceived psychological stress (2).

Additionally, Our meta-analysis identified a significant potential for publication bias, indicated by a noticeable asymmetry in the funnel plot and confirmed by a highly significant result from Egger's regression test (*p* < 0.0001). This finding implies the possibility of small-study effects or selective publication, where smaller studies with non-significant or negative findings may be underrepresented in the published literature and in the meta-analysis.

The observed publication bias suggests that the actual impact of psychological stress on stroke risk could be smaller than what the results show, especially since studies with little or no association may not have been published or included. This bias reflects a broader challenge in scientific publishing, where studies with statistically significant results are often more likely to be published, which can skew the overall evidence base. Despite this possibility for inflation, the consistent direction of the effect across multiple studies still carry a considerable weight to the conclusion that psychological stress is a risk factor for stroke.

## Clinical and practical applicability of findings

Findings in our meta-analysis highlights the importance of psychological stress as a modifiable risk factor for stroke. These findings suggest that clinical protocols for stroke prevention need to incorporate more than the traditional focus of cardiovascular risk factors and consider a systematic assessment and management of psychological stress. The numerically stronger association observed in women, even if not statistically significant in subgroup analysis, warrants specific attention. It suggests that stress management interventions might be particularly beneficial or need to be tailored for female patients, given findings in other studies that showed higher stress associations with stroke in women. Also, future research should investigate the specific mechanisms by which psychological stress impacts stroke severity and mortality, potentially through the examination of biomarkers related to inflammation, coagulation, or neurovascular integrity, and further explore the role of stress-related behaviors and access to healthcare in mediating these effects.

## Limitations

This meta-analysis has several limitations that should be considered when interpreting its findings.

First, there was considerable variability in how perceived psychosocial stress was measured, and a lack of a clear, specific, or standardized assessment. Additionally, no available data indicated whether stress levels remained consistent throughout the follow-up period, limiting our ability to distinguish between ongoing chronic stress and acute, short-term exposures. Furthermore, the use of single item self reported tools in some studies can affect the accuracy of the reported stress exposure leading to an increase in the risk of bias and subjectivity. Second, the observational nature of the both cohort and case-control studies can have few limitations of their own, although they show associations between stress and stroke, they can't prove a direct cause linking the two. Also, the presence of multiple confounders made it more complicated to isolate the effects of stress from other coexisting risk factors which may contribute to stroke risk.

Third, High statistical heterogeneity was observed, suggesting notable differences among study populations, methodologies, and stress definition making it difficult to achieve generalized conclusions. While we explored potential sources of heterogeneity through subgroup analyses, this variability makes it difficult to draw universally generalized conclusions from the pooled estimates alone.

Finally, the inclusion criteria, which sometimes involved selecting populations based on specific age, gender, or geographic regions, limit the overall generalizability of our findings

Restricting the inclusion criteria to studies that are only in English-language may have excluded other valuable research. Moreover, Egger's regression test showed potential publication bias, suggesting that smaller or non-significant studies might have been underrepresented, which could result in artificially higher pooled results.

## Strengths

Key strengths of this meta-analysis are the inclusion of a large sample of over 950,000 individuals which enhances both the generalizability and statistical reliability of the results. Furthermore, an extensive literature search was conducted across multiple databases, which was crucial in limiting the omission of relevant studies.

Another notable strength was the extensive adjustment for multiple confounders within the included studies, such as age, BMI, smoking, hypertension, diabetes, alcohol consumption, physical activity, blood lipids, and socioeconomic indicators. This adjustment increases confidence in establishing an independent association between stress and stroke, supporting that the association observed is not an artifact of differences in health characteristics or demographics.

## Future directions

To further advance the understanding of the relationship between psychological stress and stroke, future research should aim to address the limitations identified in this meta-analysis. Specifically, developing Standardized, consistent, validated tools for measuring perceived psychosocial stress are essential. as this will help researchers in achieving valid comparisons and identifying aspects of stress associated with the increased risk of stroke. Implementing regular and repeated measurements of perceived stress throughout the follow-up period, rather than a single baseline measurement would provide valuable insight into whether chronic, cumulative, or fluctuating stress exposure is most strongly associated with stroke occurrence. Further Broader inclusion criteria will help in highlighting cultural differences in how psychosocial stress is experienced and its effects on health thereby increasing the generalizability and relevance of future research findings.

## Conclusion

Our meta-analysis confirms a consistent association between psychosocial stress and stroke incidence, with prospective studies showing approximately a 50% higher risk and case–control studies supporting this relationship. Although the increased risk was observed in both women and men, the slightly higher—but not statistically significant—odds among women warrant further investigation. Importantly, while our analysis also indicated an association between higher stress levels and fatal stroke events, this result should be interpreted cautiously due to the small number of available studies contributing to that estimate and the potential influence of publication bias. Overall, these findings emphasize the need to incorporate stress assessment and management into comprehensive stroke prevention and public health strategies.

## Data Availability

The original contributions presented in the study are included in the article/supplementary material, further inquiries can be directed to the corresponding author.
